# Electroactive Microbes Short-Circuit the Passive Film to Corrode Stainless Steel

**DOI:** 10.34133/research.1185

**Published:** 2026-03-06

**Authors:** Yuting Jin, Qin Cheng, Dake Xu, Derek R. Lovley

**Affiliations:** ^1^ Electrobiomaterials Institute, Key Laboratory for Anisotropy and Texture of Materials (Ministry of Education), Northeastern University, Shenyang 110819, China.; ^2^ Institute of Materials Intelligent Technology, Liaoning Academy of Materials, Shenyang 110004, China.; ^3^ State Key Laboratory of Digital Steel, School of Materials Science and Engineering, Northeastern University, Shenyang 110819, China.; ^4^ College of Medicine and Biological Information Engineering, Northeastern University, Shenyang, China.; ^5^ Department of Microbiology, University of Massachusetts, Amherst, MA, USA.

## Abstract

Electroactive microbes are uniquely capable of aggressively corroding metals like stainless steel that were once thought immune to microbial attack. This activity has been attributed to microbial destruction of the protective chromium oxide passive film on the stainless steel surface that protects the underlying Fe^0^ from corrosive agents, allowing the microbes to establish direct electrical contact with the Fe^0^ and extract electrons to support anaerobic respiration. We show here that the electroactive microbe *Geobacter sulfurreducens*, despite its high corrosive activity, is unable to physically breach the passive film. Instead, it enables biologically mediated electron transfer across an intact chromium oxide-rich layer that remains sufficiently insulating to block abiotic proton reduction. These findings challenge the prevailing assumption that electroactive microbes must directly contact Fe^0^ for corrosion and provide new guidance for the design of corrosion-resistant metals.

## Introduction

**Fig. 1.**
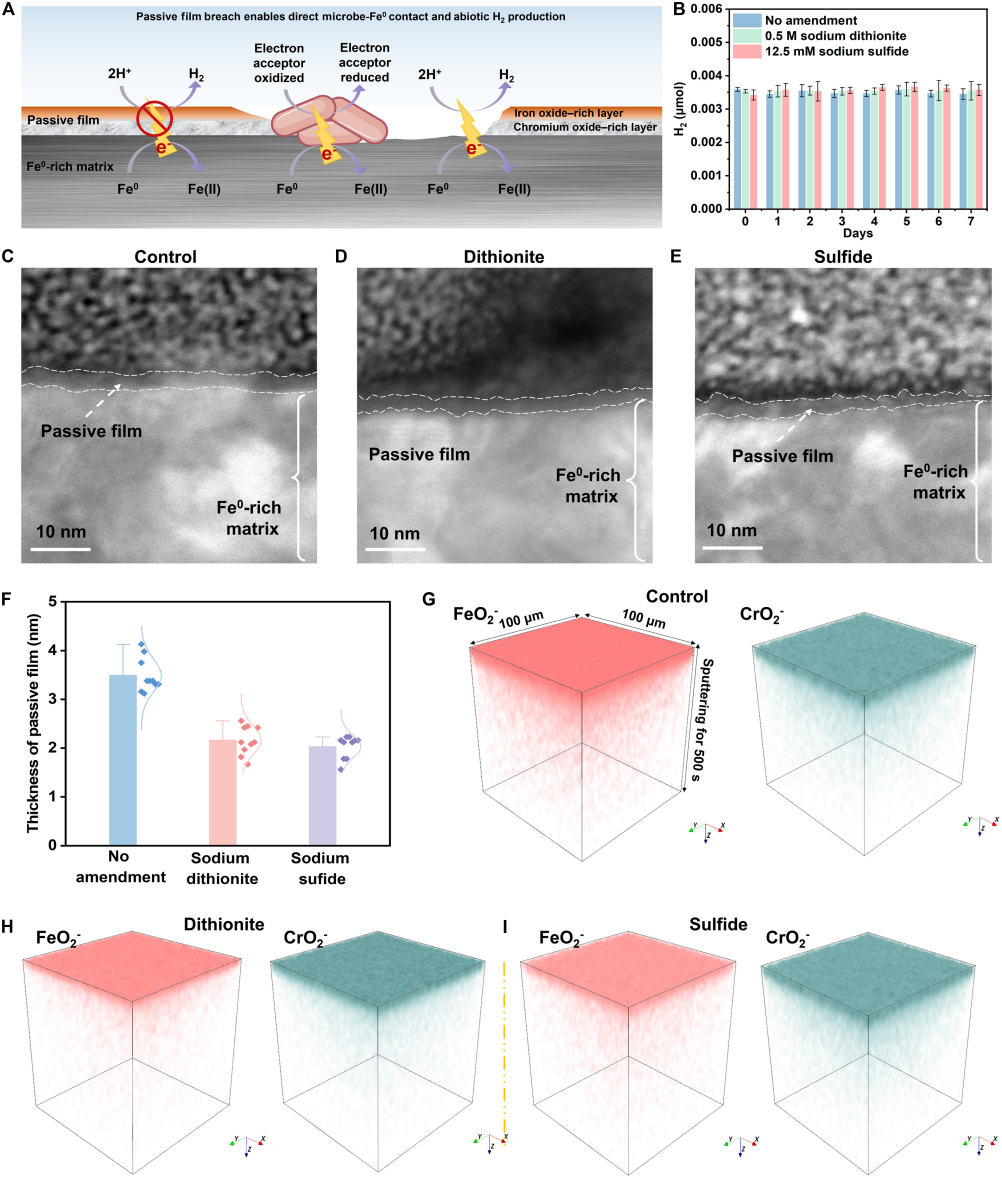
Evaluation of extracellular electron transfer model for the destruction of stainless steel passive film after 7 days. (A) Model in which electroactive microbes destroy the passive film to access the underlying Fe^0^. (B) Lack of H_2_ evolution in the presence of reducing agents (mean ± standard deviation, *n* = 3). Representative high-resolution transmission electron microscopy images for stainless steel (C) or stainless steel treated with dithionite (D) or sulfide (E). (F) Passive film thickness determined with high-resolution transmission electron microscopy (*n* = 10). Time-of-flight secondary ion mass spectrometry analysis of stainless steel (G) or stainless steel treated with (H) dithionite or sulfide (I).

One of the most deleterious impacts of microbes on modern economies is metal corrosion [1]. The innovation of stainless steel, an alloy of iron, chromium, nickel, and molybdenum, was a major advance in reducing the corrosive activity of abiotic corrosive agents (O_2_, chloride), as well as most corrosive microbes and their metabolites (sulfide and organic acids). This corrosion resistance can be attributed to the formation of a passive film that physically blocks corrosive agents from accessing the underlying metallic iron (Fe^0^) and also provides an electronic barrier to electron transfer from Fe^0^ to electron acceptors outside the passive film [2]. However, recent studies have demonstrated that electroactive microbes aggressively corrode stainless steel [3–9]. It has been widely proposed that electroactive microbes corrode stainless steel by progressively removing the iron-oxide outer layer and ultimately breaching the chromium-oxide inner layer of the passive film [2] to directly contact the underlying Fe^0^ [3,5,6,8,9] (Fig. 1A). This model is consistent with the prevailing view that the redox-active outer-surface proteins of electroactive microbes must contact extracellular electron donors and acceptors for direct electron exchange [10].

## Results and Discussion

To better understand how electroactive microbes interact with the stainless steel passive film, studies were conducted with 316L stainless steel (subsequently referred to simply as stainless steel), one of the most corrosion-resistant types of stainless steel. As previously reported [4], it did not evolve H_2_ in anaerobic incubations (Fig. 1B), demonstrating that the passive film protected the underlying Fe^0^ from reacting with protons (Fig. 1B). This contrasts with other ferrous metal forms that lack a passive film in which Fe^0^ donates electrons to protons to generate H_2_ [1]:

$${\mathrm{Fe}}^{0}+2H^{+}\to {\mathrm{Fe}}^{2+}+H_{2}$$ (1)

The passive film of the stainless steel in these incubations was readily apparent as a distinct surface layer 3.5 nm thick with high-resolution transmission electron microscopy (HR-TEM) (Fig. 1C to F).

The stainless steel was treated with the reductants dithionite and sulfide, as a simplified test to mimic the electron transfer expected from electroactive microbes. The thickness of the passive films was reduced, but a distinct, intact film remained (Fig. 1D to F). Time-of-flight secondary ion mass spectrometry (ToF-SIMS) demonstrated that the dithionite- and sulfide-induced thinning was associated with the selective removal of much of the surface iron oxide layer of the passive film, but the underlying chromium oxide-rich layer that is the key protective component of the passive film remained (Fig. 1G to I). There was also no H_2_ evolution (Fig. 1B), demonstrating that protons failed to reach the underlying Fe^0^ over the whole of the stainless steel surface. Because abiotic proton reduction on exposed Fe^0^ is rapid, the absence of H₂ evolution provides a highly sensitive, surface-integrative assay for passive-film continuity, placing a more stringent upper bound on any loss of chromium oxide barrier integrity than would be possible with localized surface-analytical measurements alone.

To determine whether some property of electroactive microbes other than serving as a source of electrons might further damage passive films, studies were conducted with *Geobacter sulfurreducens* strain ACL_HF_. *G. sulfurreducens* is the most aggressively corroding electroactive microbe known [4,7]. Strain ACL_HF_ cannot use H_2_ or formate as an electron donor, but corrodes stainless steel as fast as the wild-type strain [4]. Studies were conducted in a defined medium with fumarate as the only potential electron acceptor and stainless steel as the only potential electron donor and energy source [4].

**Fig. 2.**
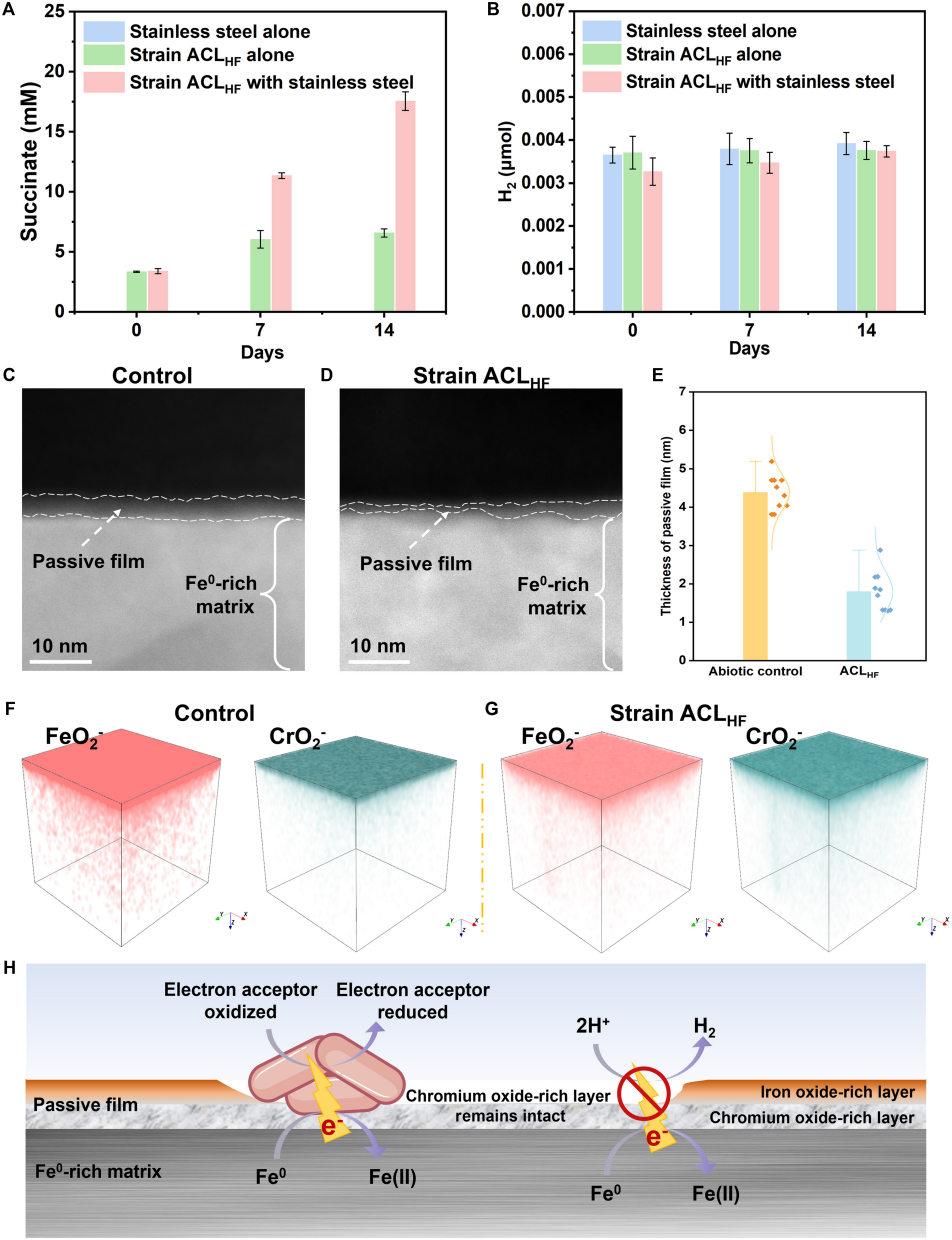
*Geobacter sulfurreducens* strain ACL_HF_ electrobiocorrosion of stainless steel. Succinate generation from fumarate reduction (A) and lack of H_2_ evolution (B) over time. High-resolution transmission electron microscopy images of stainless steel incubations without (C) or with (D) strain ACL_HF_. (E) Passive film thickness determined with high-resolution transmission electron microscopy (*n* = 10). Time-of-flight secondary ion mass spectrometry analysis of stainless steel incubations without (F) or with (G) strain ACL_HF_. (H) Model for electrobiocorrosion.

As previously reported [4], strain ACL_HF_ grew as biofilms on the stainless steel surface (Fig. S1) and reduced fumarate to succinate (Fig. 2A), which is associated with high rates of stainless corrosion [4]. HR-TEM and ToF-SIMS revealed that, like the treatments with dithionite and sulfide, strain ACL_HF_ removed a substantial quantity of the iron oxides, but the chromium oxide-rich layer remained (Fig. 2C to G). In contrast to the steady electron flux to strain ACL_HF_, there was no H_2_ production (Fig. 2B), demonstrating that the chromium oxide component of the passive film retained its integrity, preventing corrosive agents even as small as protons from reaching the underlying Fe^0^ and remaining sufficiently insulating to restrict electron transfer through the passive film to external protons for H_2_ production. Although some electroactive microbes interact with extracellular acceptors/donors with soluble electron shuttles, this possibility for electron transfer can be ruled out because (a) *G. sulfurreducens* does not produce electron shuttles; (b) even pure Fe^0^ reacts sluggishly with electron shuttles; and (c) exclusion of protons would also exclude larger shuttle molecules [10,11]. Yet, strain ACL_HF_ was able to extract electrons from the Fe^0^ underlying the passive film to support fumarate reduction.

The outer-surface redox-active proteins of electroactive microbes lower the activation energy required for oxidation or reduction at electrode surfaces, enabling oxidation/reduction reactions at much lower overpotentials [10]. Deleting genes for multi-heme *c*-type cytochromes that serve as outer-surface electrical contacts inhibits *G. sulfurreducens* stainless steel corrosion [4]. Additions of nanocrystals of magnetite, which functions like cytochromes to enhance extracellular electron exchange, accelerate it [7]. Therefore, we propose that the redox-active proteins at the microbe–passive film interface lower the activation barrier for electron transfer to the microbe below that required for abiotic H_2_ evolution. This concept is consistent with electrochemical impedance spectroscopy, which demonstrated that the charge transfer resistance between the stainless steel and *G. sulfurreducens* was ca. 9-fold lower than in the abiotic control (Figs. S2 and S3). Localized defects or thinning sufficient to permit direct microbial contact with Fe^0^ are unlikely, because such breaches would also allow proton access to the metal surface and lead to detectable H_2_ evolution, which was not observed. Instead, the findings suggest that while the passive film remains sufficiently insulating to block abiotic proton reduction, the redox activity of *G. sulfurreducens* can pull electrons from the underlying Fe^0^ across the passive film to support anaerobic respiration. This is analogous, with the direction of electron flow reversed, to the well-documented electron transfer over micrometer to millimeter scales from *Geobacter* species to electron-accepting minerals or cells of other microbial species when they are both in contact with diverse (semi-)conductive materials [10]. Determining whether this biologically activated electron transfer occurs via quantum tunneling or redox-active defect sites within the chromium-oxide lattice will require future dedicated studies beyond the scope of this Rapid Report.

These results reveal that electroactive microbes can “short-circuit” the passive film that physically and electrically protects Fe^0^ from other corrosive chemicals and microbes (Fig. 2H). Preventing electrobiocorrosion will likely require alloy or coating designs that suppress biologically activated interfacial electron transfer, not merely abiotic corrosion pathways. The discovery that microbes can drive electron flux through a material once considered electrically insulating also greatly expands the range materials that may facilitate microbe–mineral or microbe–microbe electron exchange.

## Materials and Methods

### Abiotic and microbial treatment of stainless steel

316L stainless steel was cut into square coupons (10 mm wide, 10 mm long, 4 mm thick) abraded with silicon carbide paper up to 5,000 grit, then polished to a mirror finish, ultrasonically cleaned in anhydrous ethanol for 20 min, air-dried, and sterilized under ultraviolet light for at least 30 min. For microbial corrosion studies, the coupons were added into 30 ml of the previously described fumarate-containing NB defined medium [4] in 60-ml serum bottles that were flushed with N_2_/CO_2_ (80:20 vol/vol) to remove oxygen and sealed with thick butyl rubber stoppers. The medium was inoculated with *G. sulfurreducens* strain ACL_HF_, which was obtained from our laboratory culture collection, and incubated at 30 °C. To mimic the Fe(III) oxide-reducing properties of *G. sulfurreducens* in abiotic incubations with reducing agents, the anaerobic NB medium was amended with 0.5 M sodium dithionite or 12.5 mM sodium sulfide.

### Microscopy and surface analysis

To analyze the microstructure of the stainless steel passive films, a layer of tungsten or platinum was deposited on the surface and then a cross-section of the stainless steel was milled and polished with the focused ion beam (FIB) of a Helios 5 UX or Helios G4-CXe PFIB (Thermo Fisher Scientific, USA). The cross-sectional morphology of the FIB slices was characterized with transmission electron microscopy (Talos F200X G2, Thermo Fisher Scientific, USA). The thickness of the passive film on each region of the FIB slice was statistically analyzed.

Depth profiles of the composition of the stainless steel surface were determined with ToF-SIMS (TOF.SIMS 5-100, IONTOF GmbH, Germany). A coupon surface area of 100 μm × 100 μm was analyzed with a Bi ion sputter beam operating at a voltage of 30 kV.

### Analytical techniques

For succinate determination, culture aliquots were filtered (0.22 μm pore diameter; polyvinylidene difluoride membrane) and analyzed with a high-performance liquid chromatograph (Vanquish Core, Thermo Fisher Scientific, USA). Separation was performed on a C18 column (Ultra AQ, Restek, USA) with an eluent of 1% acetonitrile and 99% 50 mM aqueous K_3_PO_4_ (pH 2.5) and detected at 210 nm. Headspace H_2_ concentrations were measured with a gas chromatograph (Trace 1310, Thermo Fisher Scientific, USA) equipped with a thermal conductivity detector. The H_2_ detection limit was 5 × 10^−6^ atm, sufficient to detect even the trace H_2_ contaminant in the N_2_/CO_2_ incubation gas mixture.

## Data Availability

All data are available from the corresponding authors upon request.
