# Supplementary material for: Electroactive Microbes Short-Circuit the Passive Film to Corrode Stainless Steel
Source: Research (Wash D C). 2026 Mar 6;9:1185. doi: 10.34133/research.1185 (PMC12963726; doi:10.34133/research.1185)
Supplement: Supplementary 1 — Supplementary Methods Figs. S1 to S3 [file research.1185.f1.zip › Supplementary Materials.docx]

Electroactive Microbes Short-Circuit the Passive Film to Corrode Stainless Steel

Yuting Jin^1,2,†^, Qin Cheng^1,3,4,†^, Dake Xu^1,3*^, and Derek R. Lovley^1,3,5*^

^1^Electrobiomaterials Institute, Key Laboratory for Anisotropy and Texture of Materials (Ministry of Education), Northeastern University, Shenyang 110819, China

^2^Institute of Materials Intelligent Technology, Liaoning Academy of Materials, Shenyang 110004, China

^3^State Key Laboratory of Digital Steel, School of Materials Science and Engineering, Northeastern University, Shenyang 110819, China.

^4^College of Medicine and Biological Information Engineering, Northeastern University, Shenyang, China

^5^Department of Microbiology, University of Massachusetts, Amherst, MA, USA

†These authors contributed equally to this work.

^*^Address correspondence to: Dake Xu; [xudake@mail.neu.edu.cn](mailto:xudake@mail.neu.edu.cn) and Derek R. Lovley; dlovley@umass.edu

**Supplementary Methods**

**Biofilm analysis**

Biofilms growing on stainless steel were imaged with scanning electron microscopy (SEM) and confocal laser scanning microscopy (CLSM). For SEM the stainless steel coupons were gently rinsed with sterile phosphate-buffered solution (pH = 7.4) and fixed with 2.5% (w/w) glutaraldehyde at 4 °C for 4 h. The samples were then dehydrated with a graded ethanol series (50%, 70%, 90%, 95%, and 100% v/v) for 8 min at each concentration. The dehydrated samples were then air-dried and sputtered with a gold film to ensure conductivity. The biofilms were examined with a EVO10 scanning electron microscope (Zeiss, Germany).

For CLSM evaluation of biofilm viability and structure the biofilms were rinsed with the phosphate buffer and then stained with the LIVE/DEAD Biofilm Viability kit (Invitrogen, Eugene, OR, USA), which contains SYTO-9 and propidium iodide (PI). After staining in the dark for 15 min., the coupons were rinsed with 0.9% NaCl solution to remove excess dye and air-dried on absorbent paper. The biofilms were observed with a model LSM900 confocal laser scanning microscope (Zeiss, Germany). The excitation wavelengths were set at 488 nm for SYTO-9 (live bacteria) and 559 nm for PI (dead bacteria). Live bacteria stain green with this procedure.

**Electrochemical impedance spectroscopy (EIS) measurement**

EIS measurements were conducted daily throughout the 7-day incubation period to monitor the electrochemical evolution at the metal-biofilm/solution interface. A sinusoidal alternating current signal with an amplitude of 5 mV was applied over a frequency range from 10^5^ to 10^−2^ Hz. subsequently fitted using an electrical equivalent circuit model *R*_s_ (*Q*_f_ (*R*_f_ (*Q*_dl_ *R*_ct_))). *R*_s_ is the resistance of the incubation solution. *Q*_f_ and *R*_f_ are the capacitance and the resistance of the biofilm and/or the corrosion product film, respectively. *Q*_dl_ and *R*_ct_ correspond to the electric double layer capacitance and the charge transfer resistance, respectively. Data fitting and parameter extraction were performed using ZSimpWin software (Version 3.30, Princeton Applied Research, USA).

**Supplementary Figures**

**
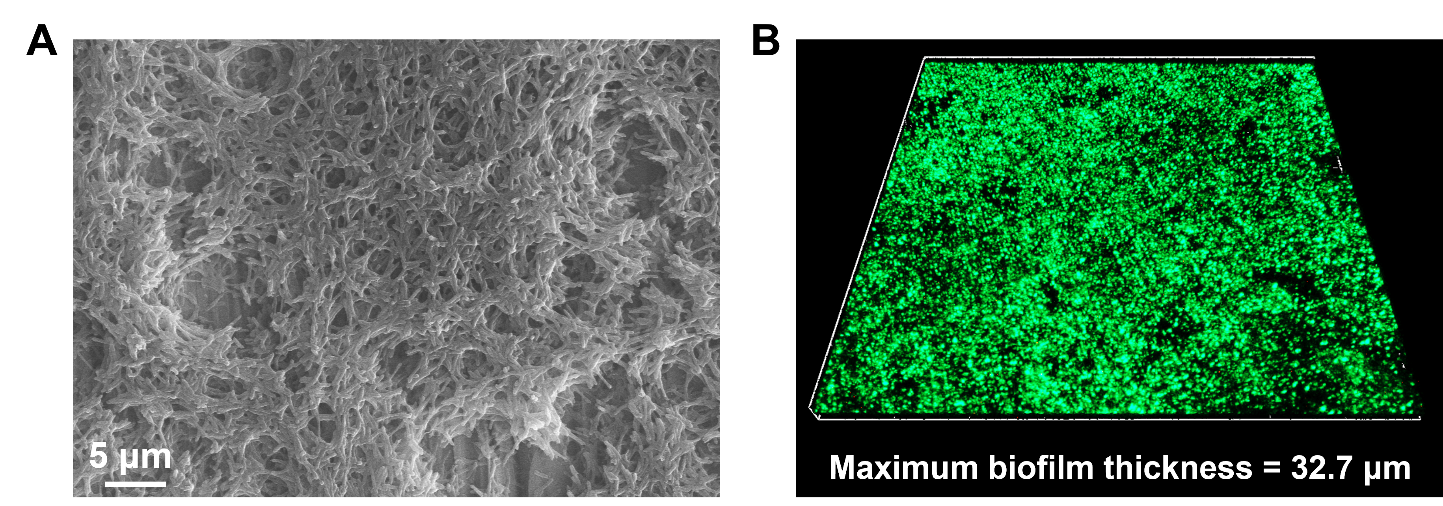
**

Fig. S1. Scanning electron microscope (A) and confocal laser scanning microscopy (B) images of strain ACL_HF_ biofilm after 7 days of incubation.


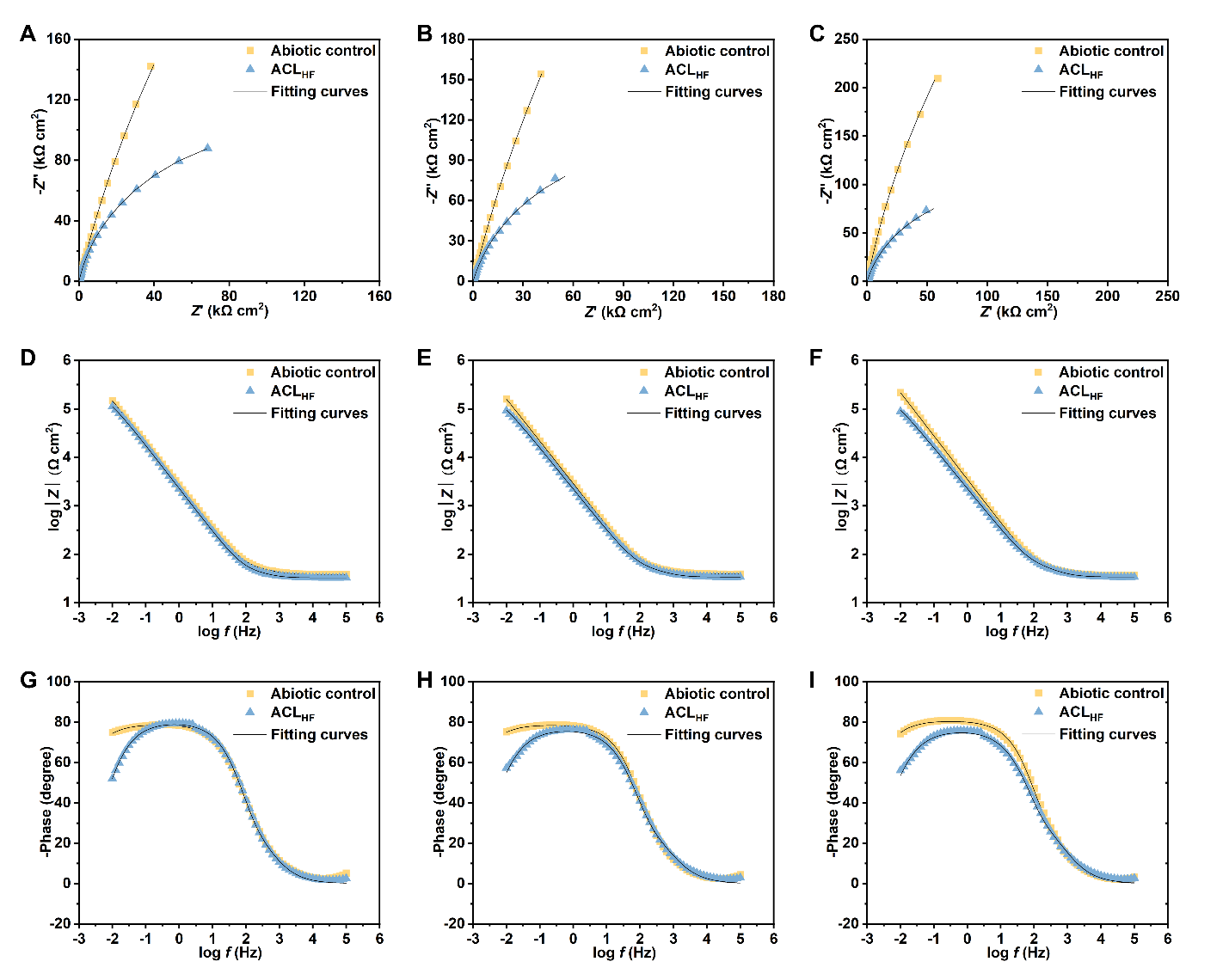


Fig. S2. Electrochemical impedance spectroscopy spectra. Nyquist plots and Bode plots for abiotic control and strain ACL_HF_. (A), (D), (G) 1 d. (B), (E), (H) 4 d. (C), (F), (I) 7 d.


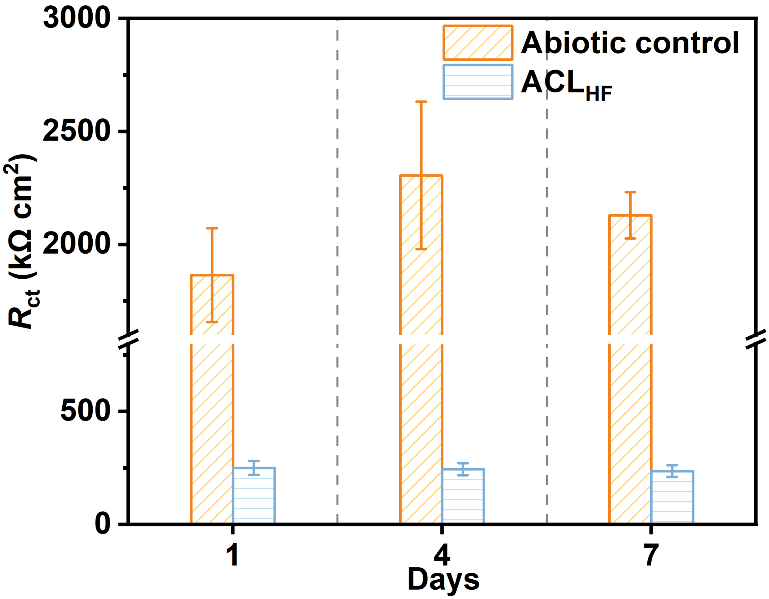


Fig. S3. Charge transfer resistance (*R*_ct_) for abiotic control and strain ACL_HF_.

As shown in the Nyquist plots (Fig. S2A–C), all spectra exhibited incomplete capacitive loops, characteristic of the capacitive behavior of the passive film on the stainless steel surface. The diameter of the capacitive loop is directly related to the polarization resistance of the material; a larger diameter indicates higher corrosion resistance. Throughout the incubation period (Days 1, 4, and 7), the capacitive loop diameters for the abiotic control were consistently and significantly larger than those for the stainless steel incubated with strain ACL_HF_. This trend indicates that the corrosion resistance of the stainless steel was substantially reduced in the presence of the bacteria.

The Bode-|Z| plots (Fig. S2D–F) further corroborate this finding. The impedance modulus at the lowest frequency (|Z|_0.01 Hz_), which is often used as a semi-quantitative indicator of corrosion resistance, was markedly lower for the strain ACL_HF_ group compared to the abiotic control at all time points. Additionally, the Bode-phase plots (Fig. S2G–I) show a broad phase angle peak for the abiotic control, suggesting a stable and protective passive film. In contrast, the phase angle curves for the strain ACL_HF_ group shifted, suggesting a change in the film's dielectric properties facilitating charge transfer.

To quantify these changes, the EIS spectra were fitted to equivalent circuits to extract the charge transfer resistance, which occurs at the metal-film interface. As shown in Fig. S3, the *R*_ct_ values remained high throughout the experiment, ranging from approximately 1800 to 2300 kΩ cm^2^, indicating that the stainless steel maintained a robust passive state in the abiotic control. The presence of the strain ACL_HF_ resulted in a dramatic decrease in *R*_ct_. The values dropped to approximately 250 kΩ cm^2^ and remained at this lower level from Day 1 through Day 7.

The substantial reduction in *R*_ct_ (by nearly an order of magnitude) in the strain ACL_HF_ system provides direct kinetic evidence that strain ACL_HF_ accelerates the corrosion of stainless steel. Since the *R*_ct_ represents the energy barrier for electron transfer across the interface, the significantly lower *R*_ct_ values in the presence of ACL_HF_ indicate that the biofilm greatly facilitates the charge transfer process, thereby accelerating the corrosion rate, despite the physical presence of the passive film.
